# Oxandrolone for treatment of lipodermatosclerosis: case report

**DOI:** 10.1590/1677-5449.190031

**Published:** 2019-10-30

**Authors:** Leonardo Amédée Péret, Heloisa Malaquias Vidal, Gabriela Alves Cardoso Gomes, Gabriel Victor Borba Oliveira, Lainara Magalhães Aguiar

**Affiliations:** 1 Universidade José do Rosário Vellano – UNIFENAS, Faculdade de Medicina, Belo Horizonte, MG, Brasil.

**Keywords:** lipodermatosclerosis, oxandrolone, venous insufficiency, case reports

## Abstract

Lipodermatosclerosis is a panniculitis characterized by hardening and hyperpigmentation of the skin involving the calves with an “inverted champagne bottle” appearance. Many therapeutic approaches have been recommended, but the use of oxandrolone for this purpose has been studied very little to date. We report a case of acute lipodermatosclerosis in a 61-year-old woman with a previous history of surgical treatment for venous insufficiency of the lower limbs. The patient presented with edema and painful, erythematous lesions with diffuse infiltration, mainly affecting the posterior aspect of the left calf. She was initially treated with stanozolol and pentoxifylline, with good response. Due to unavailability of stanozolol, she was put on oxandrolone. This treatment was well tolerated, reduced the intensity of edema, erythema, and infiltration in the lower limbs, effectively leading to pain relief. Oxandrolone may be a useful and safe treatment for patients with acute lipodermatosclerosis.

## INTRODUCTION

Lipodermatosclerosis (LDS) was described in 1955 by Huriez, who named it hypodermitis sclerodermiformis.^1^ It is a form of panniculitis affecting the lower limbs (LL), generally occurs as a complication of chronic venous insufficiency (CVI),^1^ and is more common among middle-aged or elderly women.^1^^,^ 2

Chronic venous insufficiency affects people of varying ages and can be detrimental to socioeconomic status and quality of life.^3^ The disease causes disorders of the skin and subcutaneous tissues, especially in the LL, which are the result of long-lasting venous hypertension, caused by valve incompetence and/or venous obstruction.

Sustained venous hypertension compromises venous return from the LL during exercise. This results in increased vascular permeability with release of inflammatory mediators and proteolytic enzymes, which are the underlying cause of the cutaneous hyperpigmentation, trophic cutaneous lesions, LDS, and ulceration.^3^^-^ 5

Lipodermatosclerosis is also known as sclerosing panniculitis and is characterized by hardening and hyperpigmentation of the skin of the calves, resulting in a “inverted champagne bottle” appearance.^1^^,^ 2 However, in its acute and initial form, it presents with edema, erythema, infiltration, local heat, and pain, and is often confused with erysipelas.

Elevation of the LL and compression are the two pillars of treatment for LDS.^3^^,^ 5 ^,^ 6 Good treatment results have been reported with the anabolic steroid stanozolol,^6^^,^ 7 especially at onset of the disease. Anabolic steroids increase fibrinolysis and can reduce pain, the extent of involvement, and skin hardening. However, the adverse effects, such as sodium retention, lipid profile disorders, hepatotoxicity, and virilization in women, restrict their use. Oxandrolone is an anabolic steroid with lower hepatotoxicity and androgenic effects and is another therapeutic option that has been studied little.^7^

We report a case of acute LDS in a 61-year-old woman with a prior history of surgical treatment for LL venous insufficiency. She was initially treated using stanozolol and then with oxandrolone, with good results.

This case report complies with the CARE (CAse REport) guidelines.^8^

## CASE DESCRIPTION

**Figure 1:**
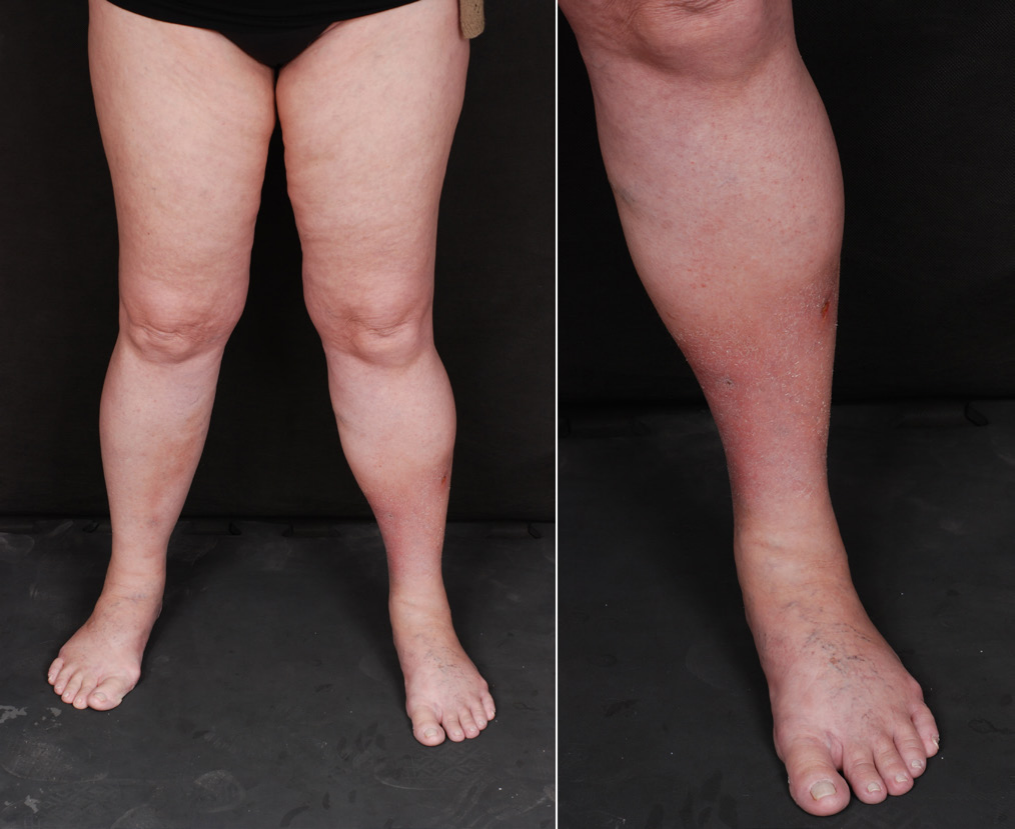
Erythema, edema, infiltration and areas of fibrosis, predominantly involving the distal part of the left lower limb.

The patient was a 61-year-old female who had undergone surgical treatments for LL venous insufficiency in 2009 and 2014. After the second surgery, she developed edema and painful erythematous lesions involving the legs, predominantly the left lower limb (LLL). She was put on systemic corticosteroids and antibiotics up to February of 2015, but did not recover completely. After the medications were withdrawn, both pain and lesions began to worsen once more. At her first consultation, in April of 2015, she presented with poorly defined subcutaneous nodules, with diffuse infiltration, primarily involving the posterior surface of the left calf. On the anterior surface of the legs, she had erythema, infiltration, scaling, and a certain degree of atrophy (Figure 1).

The patient had laboratory test results within normal limits for full blood count, erythrocyte sedimentation rate, fasting glycemia, transaminases, and gamma glutamyl transferase (GGT). Additionally, antinuclear factor was negative, chest X-ray revealed no abnormalities, tuberculin test was negative, total cholesterol was 292 mg/dL (reference value[RV]< 240 mg/dL), LDL cholesterol was 228 mg/dL (RV < 160 mg/dL), HDL cholesterol was 32 mg/dL (RV > 50 mg/dL), and triglycerides were 159 mg/dL (RV < 150 mg/dL).

A duplex scan of the LL conducted in May of 2015 showed bilateral partial great saphenectomy, bilateral segmental small saphenous vein incompetence, insufficient tributary and non-tributary veins, insufficient perforating veins, and lymphedema, while the deep vein system was patent and competent. Histopathology of the lesion revealed dermis with discrete perivascular and interstitial lymphocytic-histiocytic inflammatory infiltrate and myxoid edema. The hypodermis had septal neovascularization and sporadic foam histiocytes. In view of the history and physical examination findings, associated with the laboratory results that did not suggest a specific diagnosis of other types of panniculitis, it was concluded that the probable diagnosis was acute LDS.

In May of 2015, treatment was initiated with 2 mg of stanozolol, twice a day, and 400 mg of pentoxifylline, three times a day, obtaining significant improvement in infiltration, erythema, and pain. The patient was unable to tolerate wearing compression stockings. By September of 2015, her condition was stable, but the lesions were still symptomatic and it was necessary to increase the stanozolol dosage to 4 mg twice a day, and pentoxifylline to 800 mg, three times a day. The following month, because the condition had not resolved, topical clobetasol propionate cream was prescribed, applied with occlusive dressings. In November of 2015, the dosage of pentoxifylline was reduced to 400 mg, three times a day, because of nausea and diarrhea, with resolution of symptoms. In December, the lesions had improved significantly and the stanozolol dosage was reduced. Use of compression stockings was attempted again, but was unsuccessful once more. No lipid profile or hepatic function disorders were observed.

**Figure 2:**
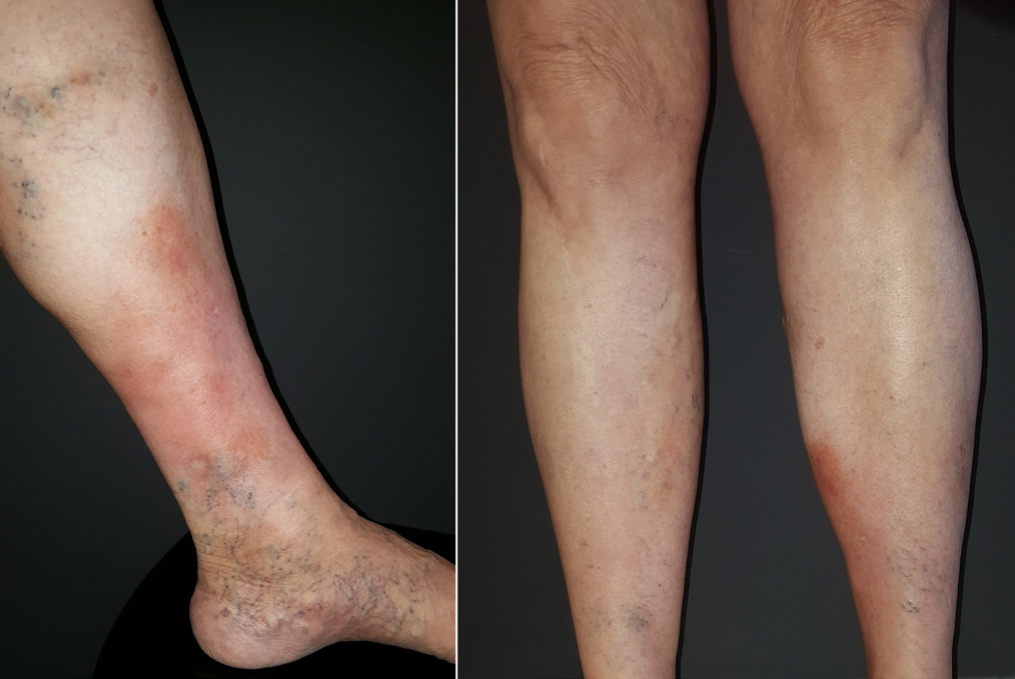
By July of 2016, there was notable improvement in the patient’s condition, with regression of erythema and infiltration in the anterolateral portion of the distal third of the left lower limb. The patient stated that pain had improved.

In April of 2016, stanozolol was suspended because it was no longer available on the market, and the patient’s lesions worsened and her pain increased. Consideration was given to substituting it with danazol, but the elevated cost was prohibitive. The decision was taken to use 10 mg of oral oxandrolone, twice a day, which resulted in significant improvements in the patient’s pain, infiltration, and erythema (Figure 2).

Oxandrolone was continued for a prolonged period, in combination with the pentoxifylline and topical clobetasol, with excellent response to the treatment. When the oxandrolone was suspended at times for financial reasons, the patient’s clinical status worsened. When it was reinitiated, symptoms improved. The patient did not exhibit any hepatic function or lipid profile disorders, maintaining a similar profile to that observed before treatment. Abdominal ultrasonography conducted at the end of 2016 showed that liver had normal dimensions, with the parenchyma free from abnormalities. No androgenic effects, such as acne, hirsutism, or changed voice were observed.

**Figure 3:**
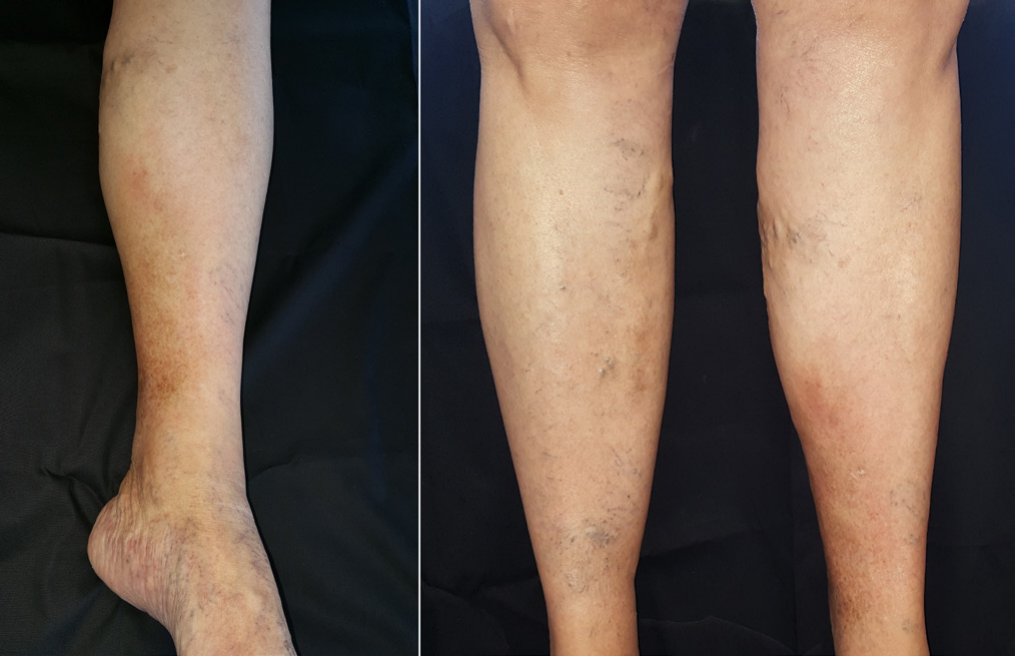
In January of 2018, it was observed that the condition had resolved completely. The patient stated that the pain had regressed totally.

In August of 2017, despite not wearing compression stockings and after withdrawal of the clobetasol and the pentoxifylline, the patient was in almost complete remission of the acute form of LDS, taking only 10 mg of oxandrolone twice a day. In January of 2018, she was well and suspended treatment for financial reasons. Since then, she has remained stable, without any type of treatment for LDS (Figure 3).

## DISCUSSION

Many therapeutic approaches have been recommended for patients with LDS, including elevation of the LL, compression therapy, hydroxychloroquine, pentoxifylline, stanozolol, and capsaicin.^7^^,^ 9 There are reports similar to this in the literature, describing cases of LDS refractory to conventional treatment with compression stockings, in which patients were unable to tolerate the pain or compression of their legs.^10^

Good results have been reported with use of anabolic steroids for treatment of LDS.^7^^,^ 11 ^-^ 13 Probably because of its fibrinolytic properties, stanozolol offers good results, despite the side effects (sodium retention, hepatotoxicity, and lipid profile abnormalities).^5^^,^ 9 ^,^ 11 ^,^ 14 A randomized, double-blind study demonstrated that use of 4 mg/day of stanozolol produces asymptomatic and temporary elevation of transaminases and depresses HDL levels in a significant proportion of patients with LDS and leg ulcers. The changes observed were reversible with withdrawal of the treatment.^11^ Dakovic et al. evaluated the clinical efficacy of stanozolol for reducing pain and dermal thickness in patients with acute LDS who were unable to sustain compression therapy.^12^ They observed that taking stanozolol for more than 8 weeks reduced pain and dermal thickness with efficacy and safety. Similarly, in the case described here, stanozolol resulted in a significant improvement in the patient’s condition, without adverse events, even after the dose was increased.

Faced with the unavailability of stanozolol, danazol can be used as a substitute,^5^^,^ 11 ^,^ 13 since they both have similar mechanisms of action. In the literature, there is one case of LDS in which administration of danazol significantly reduced pain and hardening of skin.^13^ Although effective, the high cost of this drug was the reason for choosing to use oxandrolone in the case described here.

Our report demonstrates that oxandrolone can be an effective agent for pain relief and for reducing infiltration and erythema, without changing the lipid profile or hepatic enzymes. Along the same lines, Segal et al.^7^ reported a case of chronic LDS in which use of stanozolol was discontinued because of elevation of hepatic transaminases. After substitution with oxandrolone, pain and skin hardness reduced, without provoking abnormal levels of transaminases. It is believed that oxandrolone may be less virilizing than other androgens.^5^^,^ 15

Pentoxifylline has also proven useful for treatment of LDS, through prevention of damage to the vascular endothelium.^9^^,^ 14 One study demonstrated that use of 800 mg pentoxifylline, three times a day, as an adjuvant treatment to compression therapy was more effective than a combination with placebo for treatment of chronic venous ulcers of the LL. Gastrointestinal side effects (nausea, diarrhea) were common, as in this report, primarily at a dosage of 800 mg, three times a day.^16^ Choonhakarn and Chaowattanapanit^9^ conducted a retrospective study in which they observed regression of pain, erythema, edema, and hardening of LL in patients with LDS treated with hydroxychloroquine and pentoxifylline. Along the same lines, pentoxifylline was also used as a complementary therapy in the present case.

Oxandrolone as a substitute for stanozolol in treatment for LDS, combined with pentoxifylline, was well-tolerated and resulted in clinical improvement. In view of these findings and the published data, it is believed that oxandrolone may be a good therapeutic option for cases of acute LDS that respond to stanozolol, in which hepatotoxicity sets in or when stanozolol is not available.

Since it is a case report, this study is limited. It is important that larger studies be conducted that can indicate the best therapeutic management in these cases, since LDS is a condition that is significantly detrimental to patients’ quality of life.
