# Supplementary material for: Oxandrolone for treatment of lipodermatosclerosis: case report
Source: J Vasc Bras. 2019 Oct 30;18:e20190031. doi: 10.1590/1677-5449.190031 (PMC6830406; doi:10.1590/1677-5449.190031)
Supplement: Translation

# Translation: Oxandrolona no tratamento da lipodermatoesclerose: relato de caso

**Authors:** Leonardo Amédée Péret, Heloisa Malaquias Vidal, Gabriela Alves Cardoso Gomes, Gabriel Victor Borba Oliveira, Lainara Magalhães Aguiar

**Affiliations:** 1 Universidade José do Rosário Vellano – UNIFENAS, Faculdade de Medicina, Belo Horizonte, MG, Brasil.

**Keywords:** paniculite, oxandrolona, insuficiência venosa, relatos de casos

## Abstract

A lipodermatoesclerose é uma paniculite que se caracteriza por endurecimento e hiperpigmentação da pele envolvendo as panturrilhas, com a aparência de “garrafa de champanhe invertida”. Muitas abordagens terapêuticas têm sido recomendadas, mas o uso de oxandrolona para essa finalidade foi pouco estudado até o momento. Relatamos um caso de lipodermatoesclerose aguda em uma mulher de 61 anos, com história prévia de tratamento cirúrgico para insuficiência venosa de membros inferiores. A paciente apresentava edema e lesões dolorosas e eritematosas com infiltração difusa, que acometiam principalmente a face posterior da panturrilha esquerda. Foi tratada inicialmente com estanozolol e pentoxifilina, com boa resposta. Devido à indisponibilidade do estanozolol, iniciou-se o uso de oxandrolona. Esse tratamento foi bem tolerado, resultando em redução significativa do edema, do eritema e da infiltração presentes nos membros inferiores, além de alívio da dor. A oxandrolona pode representar uma opção útil e segura no tratamento da lipodermatoesclerose aguda.

## INTRODUÇÃO

A lipodermatoesclerose (LDE) foi descrita em 1955 por Huriez, que a denominou hipodermite esclerodermiforme^1^. É uma paniculite que acomete os membros inferiores (MMII) e, geralmente, ocorre como complicação da insuficiência venosa crônica (IVC)^1^, sendo mais comum em mulheres de meia-idade ou idosas^1^^,^ 2.

A IVC acomete pessoas de diferentes faixas etárias, podendo causar prejuízos socioeconômicos e diminuição da qualidade de vida^3^. Essa doença promove alterações na pele e no tecido subcutâneo, especialmente nos MMII, que resultam da hipertensão venosa de longa duração, causada por insuficiência valvar e/ou obstrução venosa.

A hipertensão venosa sustentada compromete o retorno venoso dos MMII durante o exercício. Isso resulta em aumento da permeabilidade vascular com liberação de mediadores inflamatórios e enzimas proteolíticas, que são a causa fundamental de hiperpigmentação cutânea, alterações tróficas cutâneas, LDE e ulceração^3^^-^ 5.

A LDE é também conhecida como paniculite esclerosante e caracteriza-se por endurecimento e hiperpigmentação da pele envolvendo as panturrilhas, conferindo a aparência de “garrafa de champanhe invertida”^1^^,^ 2. Entretanto, na sua forma aguda e inicial, cursa com edema, eritema, infiltração, calor local e dor, sendo frequentemente confundida com a erisipela.

A elevação dos MMII e a compressão são os pilares do tratamento da LDE^3^^,^ 5 ^,^ 6. Bons resultados foram relatados com o esteroide anabólico estanozolol^6^^,^ 7, especialmente no início da doença. Esteroides anabólicos aumentam a fibrinólise e podem reduzir a dor, a extensão do envolvimento e o endurecimento da pele. Entretanto, os efeitos adversos, como retenção de sódio, alterações do perfil lipídico, hepatotoxicidade e virilização das mulheres, restringem o uso. A oxandrolona, esteroide anabólico de menores hepatotoxicidade e efeitos androgênicos, representa outra opção terapêutica pouco estudada^7^.

Relatamos um caso de LDE aguda em uma mulher de 61 anos, com história prévia de tratamento cirúrgico para insuficiência venosa de MMII. Ela foi tratada inicialmente com estanozolol e, posteriormente, com oxandrolona, com bons resultados.

Este relato de caso seguiu as diretrizes CARE (CAse REport)^8^.

## DESCRIÇÃO DO CASO

Uma paciente do sexo feminino, de 61 anos, foi submetida a tratamentos cirúrgicos para insuficiência venosa de MMII em 2009 e em 2014. Após a segunda cirurgia, evoluiu com edema e lesões dolorosas e eritematosas nas pernas, predominantemente no membro inferior esquerdo (MIE). Fez uso de corticosteroides sistêmicos e antimicrobianos até fevereiro de 2015, mas com melhora apenas parcial. Após a suspensão dos medicamentos, evoluiu com piora da dor e das lesões. Na primeira consulta, em abril de 2015, exibia nódulos subcutâneos mal delimitados, com infiltração difusa, acometendo principalmente a face posterior da panturrilha esquerda. Na face anterior das pernas, havia eritema, infiltração, descamação e certo grau de atrofia (Figura 1).

**Figura 1:** Eritema, edema, infiltração e áreas de fibrose predominando na parte distal do membro inferior esquerdo.

Nos exames laboratoriais, a paciente apresentava hemograma, velocidade de hemossedimentação, glicemia de jejum, transaminases e gama glutamil transferase (GGT) dentro dos valores de normalidade. Também apresentava fator antinuclear não reagente, radiografia de tórax sem anormalidades, prova tuberculínica (PPD) com resultado não reator, colesterol total de 292 mg/dL (valor de referência – VR < 240 mg/dL), colesterol LDL de 228 mg/dL (VR < 160 mg/dL), colesterol HDL de 32 mg/dL (VR > 50 mg/dL) e triglicerídeos de 159 mg/dL (VR < 150 mg/dL).

O *duplex scan* dos MMII, realizado em maio de 2015, mostrava safenectomia magna parcial bilateral, incompetência segmentar da veia safena parva bilateralmente, ramos tributários e não tributários insuficientes, perfurantes insuficientes e linfedema, além de sistema venoso profundo pérvio e competente. O exame histopatológico da lesão apresentava derme com discreto infiltrado inflamatório linfo-histiocitário perivascular e intersticial e edema mixoide. Na hipoderme, havia neovascularização septal e raros histiócitos espumosos. Diante da história e do exame físico, associados aos resultados laboratoriais, que não sugeriram diagnóstico específico de outras paniculites, concluiu-se pelo diagnóstico provável de LDE aguda.

Em maio de 2015, foi iniciado o tratamento com estanozolol 2 mg, duas vezes ao dia, e pentoxifilina 400 mg, três vezes ao dia, obtendo-se melhora significativa da infiltração, do eritema e da dor. A paciente não tolerou o uso de meias compressivas. Em setembro de 2015, exibia quadro estável, porém ainda com lesões sintomáticas, sendo necessário aumentar a dose do estanozolol para 4 mg, duas vezes ao dia, e da pentoxifilina para 800 mg, três vezes ao dia. No mês seguinte, devido à persistência do quadro, iniciou-se o uso tópico de pomada de propionato de clobetasol, aplicada com oclusão. Em novembro de 2015, reduziu-se a dose da pentoxifilina para 400 mg, três vezes ao dia, devido à ocorrência de náuseas e diarreia, com resolução dos sintomas. Em dezembro, com a melhora significativa das lesões, foi reduzida a dose do estanozolol. Reiniciou-se o uso de meias compressivas, novamente sem sucesso. Não foram observadas alterações no perfil lipídico e na função hepática.

Em abril de 2016, interrompeu-se o uso do estanozolol devido à falta do medicamento no mercado e, então, a paciente apresentou piora das lesões no MIE e da dor. Considerou-se a substituição por danazol, impossibilitada pelo custo elevado. Optou-se pelo uso oral de oxandrolona 10 mg, duas vezes ao dia, que levou à melhora significativa da dor, da infiltração e do eritema (Figura 2).

**Figura 2:** Em julho de 2016, observou-se melhora do quadro com regressão do eritema e infiltração na porção anterolateral do terço distal do membro inferior esquerdo. A paciente referiu melhora da dor.

A oxandrolona foi mantida por longo período, associada à pentoxifilina e ao clobetasol tópico, com excelente resposta terapêutica. Em alguns momentos em que a oxandrolona foi descontinuada por motivos financeiros, houve piora do quadro clínico. A reintrodução foi seguida de melhora dos sintomas. A paciente não apresentou alterações na função hepática e no perfil lipídico, mantendo padrão semelhante ao apresentado antes do tratamento. Uma ultrassonografia abdominal realizada no final de 2016 mostrou fígado com dimensões normais e parênquima sem alterações. Não se observaram efeitos androgênicos como acne, hirsutismo ou alterações da voz.

Em agosto de 2017, mesmo sem o uso das meias compressivas e após a retirada do clobetasol e da pentoxifilina, a paciente apresentava remissão quase total da forma aguda da LDE, usando somente oxandrolona 10 mg, duas vezes ao dia. Em janeiro de 2018, encontrava-se bem e suspendeu o tratamento por motivos financeiros. Desde então, permanece estável, sem nenhum tratamento para LDE (Figura 3).

**Figura 3:** Em janeiro de 2018, observou-se melhora completa do quadro. A paciente referiu regressão total da dor.

## DISCUSSÃO

Muitas abordagens terapêuticas têm sido recomendadas para pacientes com LDE, incluindo elevação dos MMII, terapia de compressão, hidroxicloroquina, pentoxifilina, estanozolol e capsaicina^7^^,^ 9. Há alguns relatos semelhantes a este na literatura de casos de LDE refratários ao tratamento convencional com meias compressivas, em que os pacientes são incapazes de tolerar a dor ou a compressão nas pernas^10^.

Bons resultados foram relatados com o uso de esteroides anabólicos no tratamento da LDE^7^^,^ 11 ^-^ 13. Devido provavelmente às suas propriedades fibrinolíticas, o estanozolol apresenta bons resultados, apesar dos efeitos colaterais (retenção de sódio, hepatotoxicidade e anormalidades no perfil lipídico)^5^^,^ 9 ^,^ 11 ^,^ 14. Um estudo randomizado e duplo-cego demonstrou que o uso de estanozolol 4 mg/dia produz elevação assintomática e temporária das transaminases e depressão do nível de HDL em uma proporção significativa de pacientes com LDE e úlceras de perna. Essas alterações encontradas foram reversíveis após a cessação do tratamento^11^. Dakovic et al. avaliaram a eficácia clínica do estanozolol para redução da dor e espessura dérmica em pacientes com LDE aguda incapazes de manter a terapia de compressão^12^. Observou-se que o uso de estanozolol por mais de 8 semanas diminuiu a dor e a espessura dérmica com eficácia e segurança. De maneira semelhante, no caso relatado, o uso de estanozolol proporcionou melhora significativa do quadro, sem eventos adversos, mesmo após o aumento da dose.

Diante da indisponibilidade do uso de estanozolol, o danazol pode atuar como substituto^5^^,^ 11 ^,^ 13, pois os dois possuem mecanismos de ação semelhantes. Na literatura, há um caso de LDE em que a administração de danazol reduziu significativamente a dor e o endurecimento da pele^13^. Apesar de eficaz, o custo elevado desse medicamento foi o motivo pelo qual se optou pela utilização de oxandrolona no caso descrito.

Nosso relato demonstrou que a oxandrolona pode ser um agente eficaz no alívio da dor e na redução da infiltração e do eritema, sem alterar o perfil lipídico e as enzimas hepáticas. De maneira semelhante, Segal et al.^7^ relataram um caso de LDE crônica cujo uso de estanozolol foi descontinuado devido à elevação das transaminases hepáticas. Após a substituição por oxandrolona, a dor e o endurecimento da pele diminuíram, sem anormalidades das transaminases. Acredita-se que a oxandrolona possa ser menos virilizante que outros andrógenos^5^^,^ 15.

A pentoxifilina também demonstra ser útil no tratamento da LDE, através da prevenção de danos ao endotélio vascular^9^^,^ 14. Um estudo demonstrou que o uso de pentoxifilina 800 mg, três vezes ao dia, como tratamento adjuvante à terapia de compressão foi mais eficaz do que a combinação com placebo no tratamento da úlcera venosa crônica dos MMII. Efeitos colaterais gastrointestinais (náusea, diarreia) foram comuns, assim como neste relato, principalmente na dose de 800 mg, três vezes ao dia^16^. Choonhakarn e Chaowattanapanit^9^, em um estudo retrospectivo, observaram regressão da dor, do eritema, do edema e do endurecimento dos MMII de pacientes com LDE tratados com hidroxicloroquina e pentoxifilina. Da mesma forma, a pentoxifilina foi utilizada neste caso como terapia associada.

A oxandrolona em substituição ao estanozolol no tratamento de LDE, associada à pentoxifilina, foi bem tolerada, resultando em melhora clínica. Diante desses achados e dos dados da literatura, acredita-se que a oxandrolona possa representar uma boa opção terapêutica para casos de LDE aguda, responsiva ao estanozolol, nos quais a hepatotoxicidade se desenvolve ou quando há indisponibilidade do medicamento.

Tratando-se de um relato de caso, este estudo é limitado. Portanto, é importante que sejam realizados estudos amplos que esclareçam o melhor manejo terapêutico nesses casos, pois a LDE é uma afecção que prejudica de maneira relevante a qualidade de vida dos pacientes.
